# On the tenth value distance of the photon field along the maze of high‐energy linear accelerator vaults

**DOI:** 10.1002/acm2.12250

**Published:** 2018-01-18

**Authors:** Zhaohui Han, Lee M. Chin

**Affiliations:** ^1^ Department of Radiation Oncology Brigham and Women's Hospital and Dana Farber Cancer Institute Harvard Medical School Boston MA USA

**Keywords:** capture gamma ray, maze, shielding, tenth value distance or TVD

## Abstract

There is a wide range in the reported photon tenth value distance (TVD) in the maze of high‐energy linear accelerator vaults. In order to gain insight into the appropriate use of the TVD value during door design, we performed measurements of the photon dose in the maze of four vaults. In addition, our study represents the first to describe a scenario where an inner borated polyethylene (BPE) door for neutron shielding is installed in the maze downstream to Point A, the point on the maze centerline that is just visible from the isocenter. The measurements were made along the maze centerline at 1 m above the floor. In all cases, the accelerator operated at a nominal energy of 15 MV. Of the four vaults, three were equipped with an inner BPE door at a distance of 1.0–2.1 m downstream to Point A. The door was made of 10.16 cm (4″) BPE sandwiched between two 0.635 cm (1/4″) steel face plates. The photon dose in the maze without a BPE door decreases exponentially with a characteristic TVD of 6 m beyond a distance of 2.5 m from Point A. The presence of a BPE door in an identical vault not only reduces the photon intensity in the maze by about an order of magnitude, but also softens the energy spectrum with a shortened TVD of 4.7 m, significantly lessening the shielding burden at the outer maze entrance. In contrast to the common use of Point A as the reference point to specify distance, the photon dose in the maze with a BPE door located downstream to Point A can be satisfactorily described as exponential functions of the distance measured from the door, which shows good consistency among the three vaults of different room parameters.

## 1 INTRODUCTION

For shielding design of a high‐energy (>10 MV) linear accelerator vault with a maze, the standard approach describes the photon dose in the maze as exponential functions of the distance along the maze centerline.^1^, ^2^, ^3^ The photon components of various origins/energies are characterized by different tenth value distances (TVD), which can be derived based on radiation measurements made in the maze.^3^ In general, the low‐energy scattered x rays have a short TVD and decrease relatively fast, posing less challenge to the door shielding.^4^ On the other hand, the capture gamma rays, which are produced through the interactions of the photoneutrons with concrete, have a relatively long TVD.^4^ Consequently, the predominant photon contribution at the outer maze entrance comes from the capture gamma rays when the maze length is greater than three meters.^4^ In addition, capture gamma rays are very energetic with an average energy of 3.6 MeV and have a tenth value layer (TVL) of 6.1 cm of lead, which could require significant amount of shielding materials resulting in a massive door.^1^

**Figure 1:**
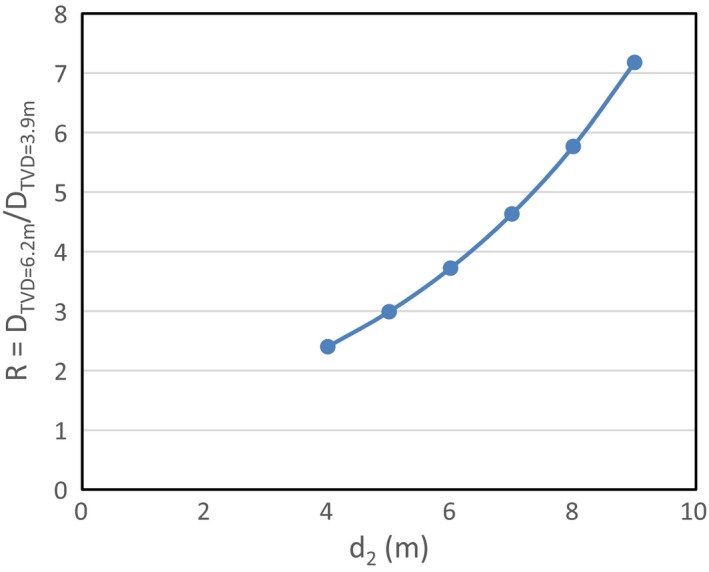
The ratio of the estimated photon dose at the outer maze entrance using a TVD value of 6.2 m (D_TVD__=6.2 m_) and 3.9 m (D_TVD__=3.9 m_) as a function of the maze length d_2_. The estimated photon dose is very sensitive to the TVD values used in the standard exponential description of photon field along the maze for long mazes.

During door design, it is essential to have a reasonably good estimate of the photon dose at the outer maze entrance in order to determine the thickness of the materials, which are usually lead and/or steel (In addition, 5% borated polyethylene or BPE is typically used in the door for neutron shielding). From a clinical point of view, a light door is beneficial because of the opening/closing speed and safety reasons. Therefore, it is desirable to put as little materials as possible in the door while achieving adequate shielding capability. This effort, however, can be largely hindered if large uncertainty exists in estimating the photon dose at the door. It is the authors’ experience that this estimate is particularly sensitive to the TVD values, which range from 3.9 to 6.2 m in the literature for a nominal energy of 15 MV.^1^, ^2^, ^5^ To better illustrate the sensitivity to TVD, we compare in Fig. 1 the photon doses calculated using a TVD value of 6.2 m (D_TVD = 6.2 m_) and 3.9 m (D_TVD = 3.9 m_), respectively. Figure 1 shows the ratio (R = D_TVD = 6.2 m_/D_TVD = 3.9 m_) as a function of d_2_, the distance between the outer maze entrance and Point A, which is defined as the point on the maze centerline that is just visible from the isocenter.^1^ As can be seen, depending on the choice of TVD values, the calculated photon dose can vary by a factor of seven for a nine‐meter‐long maze, which would result in a vastly different door design.

In a recent design of a linac vault to house a 15 MV accelerator at our institute, the large range of the reported TVD values has created a great deal of uncertainty in determining the lead thickness to be used in the door. On the one hand, assuming a TVD value of 3.9 m, ¼” of lead‐equivalent‐thick materials (in addition to 4″ BPE for neutron shielding) will suffice the institutional ALARA (As Low As Reasonably Achievable) goal of 1 mR/week outside the door. On the other hand, an additional 2″ thick lead is required if a TVD value of 6.2 m is assumed, which represents an increase of about 5,000 lbs in weight for the door measuring 87″ × 68″. Such a heavy door not only slows down the operational speed and increases the cost, but also creates engineering difficulties and potentially leads to safety issues. The lack of more precise data in TVD value in the literature has prompted us to carry out a detailed measurement in the maze once the linac was installed.

McGinley et al. have studied a number of methods that aim to replace the heavy door at the outer maze entrance, including reducing the inner maze opening size and adding neutron absorbing materials at the inner maze entrance.^6^ In all of the methods, it was found that the photon dose in the maze can be described by the sum of two exponential functions of the distance along the maze centerline, measured from Point A. However, for reasons such as clinical usage of the wall space, it might not always be feasible to place a neutron door at the inner maze entrance. At our institute, a BPE door is often placed 1–2 m downstream from Point A in the maze where the space is largely empty and unused. The different location of the inner maze door in relation to Point A, compared with that described by McGinley et al., could result in a different photon field in the maze. The BPE door is effectively a neutron absorber and capture gamma photon emitter. With the photon emitting BPE door being located downstream to Point A, it may no longer be valid to describe the radiation dose in the maze as if it “virtually” emanates from Point A.

The purposes of the study are two folds. Firstly, we provide new information of the TVD for a maze that does not incorporate an inner neutron door in an attempt to clarify the application of the reported TVD values. Secondly, we have systematically studied the photon dose in the maze in the presence of an inner BPE door that is located at 1.0–2.1 m downstream to Point A. To the best of our knowledge, there has not been a study of such a scenario in the literature where the description of the photon dose requires a new model, in contrast to that reported by McGinely et al. for inner BPE doors located upstream to Point A.^6^ In addition, two bunkers (one with an inner BPE door and the other without) in the current study have identical dimensions, which offers us unique opportunity to conduct a comparative study as the differences observed can be unambiguously attributed to the presence of the inner BPE door.

## 2 MATERIALS AND METHODS

### 2.A Vault layout

**Figure 2:**
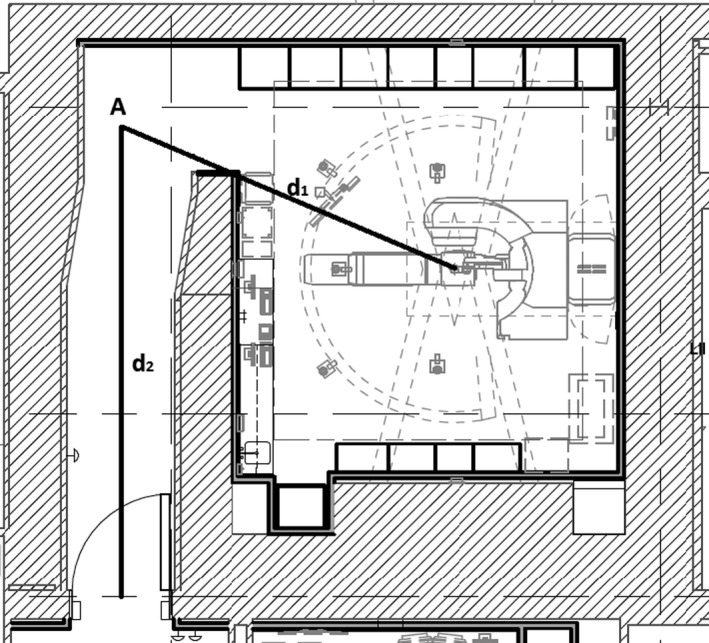
Schematic layout of the treatment rooms and mazes. Also shown are the Point A, distances d_1_ and d_2_ as defined by the NCRP Report No. 151.^1^ In addition, for Vaults 2–4, an inner door has also been installed in the maze at 1.0 to 2.1 meters downstream to Point A. The inner door is mainly for neutron shielding and consists of 10.16 cm (4″) BPE sandwiched between two 0.635 cm (1/4″) steel face plates.

Figure 2 is the architectural plan of Vault 1 which is the only room without an inner BPE door in this study. Point A, by definition, is the point on the maze centerline which is just visible from the isocenter.^1^ d_1_ is defined as the distance between the isocenter and Point A and d_2_ between Point A and the outer maze entrance.^1^ Vault 2 has identical dimensions except that an inner door for neutron shielding has also been installed in the maze 1.5 m downstream from Point A. Figure 2 also represents the schematic layout of Vaults 3 and 4, which have different dimensions with the inner door located at various distances from Point A. The inner door for all three vaults in the current study consists of 10.16 cm (4″) BPE sandwiched between two 0.635 cm (1/4″) steel face plates.

The treatment room and maze parameters are summarized in Table 1. Vaults 1–3 are all concrete construction. Vault 4 has lead bricks on the inside surface of the ceiling primary barrier, followed by BPE and concrete. Vault 4 also has 5.08 cm (2″) of BPE lining the secondary barrier adjacent to the end of the treatment couch as well as the wall surfaces at the inner maze entrance.

**Table 1:** Treatment room and maze parameters of the vaults in the current study. The definition and notation of the parameters strictly follow the NCRP Report No. 151.^1^

Vault No.	Vault 1	Vault 2	Vault 3	Vault 4
Make	Varian	Varian	Varian	Varian
Model	TrueBeam	Trilogy	Clinac IX	Novalis TX
Nominal energy (MeV)	15	15	15	15
Maze height (m)	4.6	4.6	3.4	3.0
Maze width (m)	2.1	2.1	2.1	2.0
Maze cross‐sectional area S_1_ (m^2^)	9.8	9.8	7.2	6.0
Inner maze opening height (m)	4	4	3.4	3.0
Inner maze opening width (m)	2.4	2.4	2.6	2.7
Inner maze opening area S_0_ (m^2^)	9.7	9.7	8.7	8.4
d_1_ (m)	6.6	6.6	6.7	5.9
d_2_ (m)	8.8	8.8	7.2	5.2
Room surface area (m^2^)	245	245	232	209
Distance of BPE door from Point A (m)	No BPE door	1.5	2.1	1
Neutron fluence at Point A (10^9^ m^−2^ Gy^−1^)	4.70	4.70	4.84	–

### 2.B Photon dose measurements in the maze

Photon dose was measured along the maze centerline at about 1 m above the concrete floor. Two Victoreen model 451 (Fluke Biomedical, Everett, WA, USA) survey meters were used which had been calibrated with a ^137^Cs source. The agreement of the two meters was generally within 5% and the average readings of the two meters were taken as the measured values. The survey meters were set to operate in the integrate mode for each measurement. The positional accuracy of the meters was estimated at ±5 cm.

All measurements were taken with the linac gantry pointing toward the floor, with the tertiary multileaf collimators fully retracted and the secondary collimator closed down to a nominal minimum field size of 0.5 × 0.5 cm. For each measurement, 200 and 500 monitor units (MU) of nominal 15 MV x rays were delivered for Vault 1 and Vaults 2–4, respectively. No scattering materials were used in the beam.

The calibration of linear accelerator follows the American Association of Physicists in Medicine TG‐51 protocol.^7^ At our institute, linacs are calibrated to deliver 1 cGy/MU at the isocenter in water, at the depth of maximum dose, for a field size of 10 × 10 cm. The results in the current study were normalized to the isocenter dose under the calibration conditions.

## 3 RESULTS

**Figure 3:**
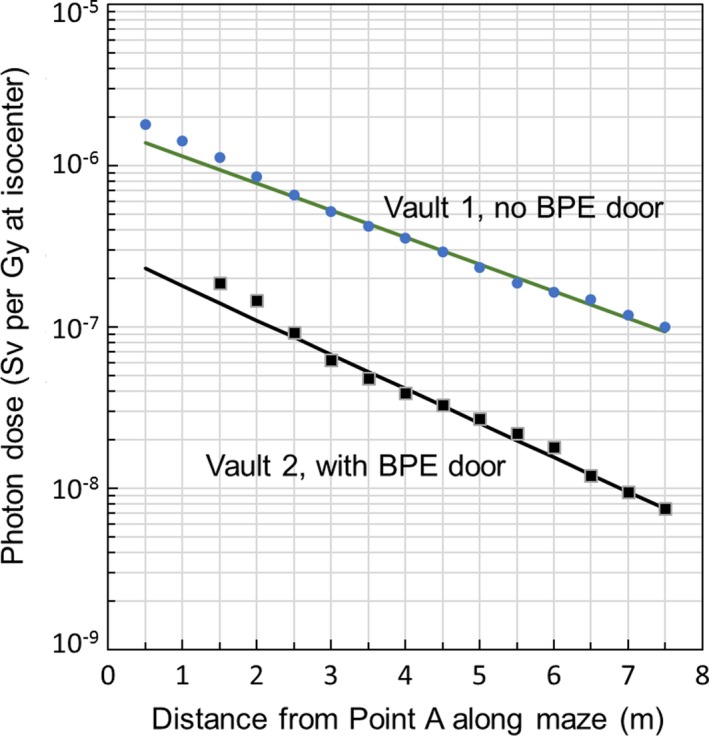
Measured photon dose in the maze for Vault 1 (circles) and Vault 2 (squares). The two vaults have identical dimensions except that Vault 2 also has an inner BPE door at 1.5 m downstream from Point A. The straight lines are exponential fits to the measurements for distances greater than 2.5 m with a characteristic TVD of 6 and 4.7 m, respectively for Vault 1 and Vault 2. The presence of the BPE door not only reduces the photon intensity in the maze by an order or magnitude but also softens the energy spectrum as evidenced by the shortened TVD.

Figure 3 shows the photon dose in the maze for Vaults 1 and 2. Both Vaults have identical dimensions; however, Vault 2 also has an inner BPE door installed at a distance of 1.5 m downstream from Point A. The symbols in Fig. 3 are measured dose (D) as a function of the distance from Point A (x) along the maze centerline. For x ≥ 2.5 m, the measurements can be fitted to an exponential function D = 1.67 × 10^−6^ × 10^−x/6.0^ for Vault 1, which is shown as a straight line in Fig. 3. Similarly, the measurements for Vault 2 can be fitted to an exponential function D = 2.93 × 10^−7^ × 10^−x/4.7^. The exponential behavior is consistent with previous reports;^1^, ^2^, ^5^ however, the derived TVD for Vault 1, which is 6.0 m, is in closer agreement with the reported 6.2 m in Ref. 5 and significantly longer than those suggested in Refs. 1 and 2. As can be seen in Fig. 3, the photon dose in the maze of Vault 2 is about an order of magnitude lower than that of Vault 1, in addition to a shortened TVD of 4.7 m.

**Figure 4:**
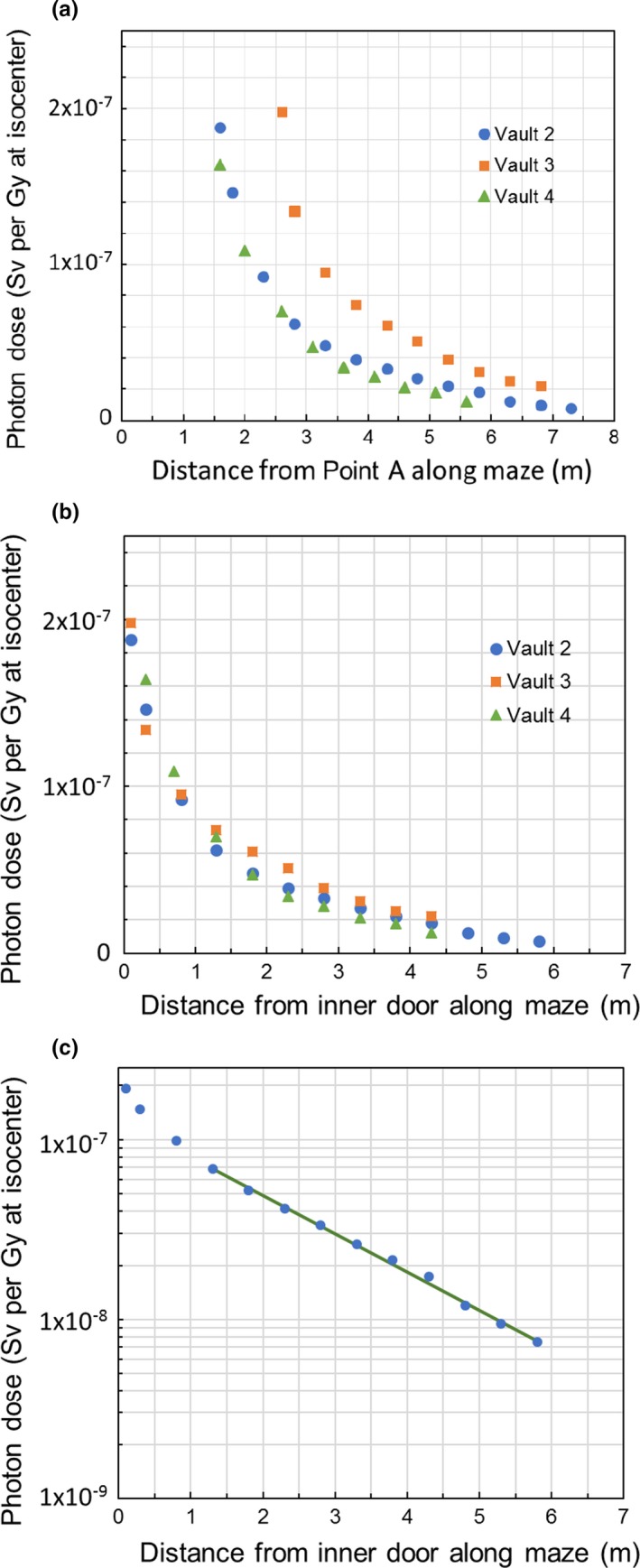
Photon dose in the maze for Vaults 2–4 in the presence of an inner door for neutron shielding: (a), measured dose on the maze centerline is shown as a function of the distance from Point A; (b), the same measured dose, when expressed as a function of the distance for the door, essentially converged to a single curve; (c), an exponential fit (solid line) to the average dose (circles) of the three vaults gives a TVD of 4.7 m.

Figure 4 shows the photon dose in the maze for Vaults 2–4 in the presence of an inner door for neutron shielding. In all three cases, the inner door is located downstream to Point A at a distance of 1.5, 2.1, and 1.0 m, respectively for Vault 2, 3, and 4. Figure 4(a) shows the measured dose as a function of the distance from Point A. When expressed as function of the distance measured from the inner BPE door, the photon dose measured on the maze centerline from all three bunkers essentially converged to a single curve as shown in Fig. 4(b). Despite the rather large difference in room parameters and the Point A–BPE door distance, Vaults 2 and 3 have very similar photon fields in the maze. In comparison, the dose decreases slightly faster for Vault 4, which might be attributable to the different materials, especially the use of BPE on the secondary barrier and on the walls at the inner maze entrance. The circles in Fig. 4(c) are the semilog plot of the average dose (D) of the three vaults as a function of the distance from the inner BPE door (y). The solid line is an exponential fit of the measurements to function D = 1.30 × 10^−7^ × 10^−y/4.7^ for y > 1 m, giving a TVD value of 4.7 m.

## 4 DISCUSSION

For a high‐energy (>10 MV) linear accelerator vault with a relatively long maze (>3 m), the photon radiation at the outer maze entrance predominately comes from the capture gamma rays as a result of the interactions of photoneutrons and the concrete surface.^4^ Compared with the softer scattered photon radiations, the capture gamma radiation poses greater challenges to the door design mainly due to two factors: (a) it is much more energetic with an average energy of 3.6 MeV and has a TVL of 6.1 cm of lead; (b) it decreases much more slowly in the maze, i.e., the TVD for capture gamma radiation is typically long.^1^, ^2^ While the first factor can be expected to be relatively constant among vaults of different design, the second may show large variations. As a result, the reported TVD values range from 3.9 to 6.2 m,^1^, ^2^, ^5^ the choice of which can lead to vastly different amount of door shielding materials as illustrated in Fig. 1.

In our newly constructed Vault 1, the derived TVD value of 6.0 m was within but closer to the upper limit of the reported range, which is in agreement with that reported by Wu et al.^5^ A closer look reveals that the apparent agreement might be attributable to the similarity in room parameters between Vault 1 of the current study and Vaults 1 and 2 of Wu et al.^5^ It is possible that the TVD can vary should the room parameters, in particular, the inner maze opening area, the maze cross‐sectional area, the isocenter–to–Point A distance, and the room surface area deviate significantly from those of Vault 1. It is important that the shielding designer thoroughly evaluate the vault dimensions before choosing a TVD value. In our case, the door would have been significantly under‐shielded had a TVD value of 3.9 m been used.

McGinley et al.^6^ has reported the use of BPE materials at the inner maze entrance (upstream to Point A) to limit the photoneutons entering the maze and therefore, reducing/eliminating the shielding materials at the outer maze entrance. In their report, the photon dose in the maze was modeled as the sum of two exponential functions of the distance from Point A. Due to clinical considerations (e.g., medical gas and storage use of the wall space), it is not always possible to install a BPE door at the inner maze entrance. At our institute, a BPE door is typically installed in the maze and usually within 2 m downstream from Point A. Intuitively, with the introduction of a BPE door, a significant photon component in the maze comes from the capture gamma rays as a result of the boron–neutron interactions in the door, i.e., the BPE door is effectively a photon radiation “source”. With the location of the BPE door (and therefore the radiation source) varying among vaults, it might no longer be valid to reference the photon distribution to a single fixed point (e.g., Point A). In particular, as the door is located downstream to Point A, the use of Point A as the reference point may not be ideal. To the best of our knowledge, our work represents the first to study such a scenario.

As can been seen in Fig. 4(a), the photon dose distribution as a function of the distance from Point A shows a rather wide range of variations among Vaults 2 to 4, for which the BPE door is located at a distance between 1.0 and 2.1 m from Point A. When expressed as a function of the distance measured from the BPE door in Fig. 4(b), the results from the three vaults essentially converge to a single curve, indicating that the location of the door is an appropriate reference point. This convergence may also suggest that the door is a dominant source of the photon dose in the maze for these vaults. Similarly to the observation by McGinley et al.,^6^ the photon field seems to consist of a “soft” component which may be attributable to the scattered x rays and decreases fast along the maze, and a “hard” component which decreases with a TVD value of 4.7 m. Interestingly, from a comparison of all concrete Vaults 2 and 3, the magnitude of the photon dose in the maze does not seem to show strong correlation with the neutron fluence at Point A and the Point A–to–BPE door distance.

It should be noted that, Vaults 1 and 2 in the current study have identical dimensions with the only difference being the existence of a BPE door in Vault 2. The incorporation of an inner BPE door not only reduces the photon dose in the maze by about an order of magnitude, but also softens the energy spectrum as evidenced by the reduced TVD value (4.7 vs 6 m). The softer radiation might be attributable to the BPE door in which the neutrons are moderated by the hydrogen abundant polyethylene and then captured by boron. The boron–neutron capture gamma ray has an energy of 478 keV,^1^ significantly lower than that from the neutron‐concrete interactions. The reduction in the number of neutrons entering the maze greatly lessens the shielding burden at the outer maze entrance. In fact, for a workload of 500 Gy/week for 15 MV, at a distance of 4.5 m from the inner BPE door, the exposure level would result in a weekly dose below our institutional ALARA goal of 1 mR/week for controlled areas without the need of a shielded door at the outer maze entrance. In contrast, a 5.08 cm (2″) lead equivalent thickness is needed (in addition to the 4″ BPE for neutron shielding) for Vault 1 to bring the radiation level outside the door to within the ALARA limit.

## 5 CONCLUSIONS

There is a wide range of the reported photon TVD value in the maze of a high‐energy linear accelerator vault. Our study has shed light on the importance of appropriate choice of the TVD value during door design. The incorporation of an inner BPE door to limit/prevent the photoneutrons from entering the maze greatly lessens the shielding burden at the outer maze entrance. Our study is the first to describe a scenario where the BPE door is located downstream to Point A. For such a case, it is found that the photon dose along the maze can be reasonably well described as exponential functions of the distance measured from the inner BPE door and is consistent among vaults of different parameters.

## CONFLICT OF INTEREST

The authors have no conflicts of interest.
